# Waist circumference thresholds predicting incident dysglycaemia and type 2 diabetes in Black African men and women

**DOI:** 10.1111/dom.14655

**Published:** 2022-02-10

**Authors:** Julia H. Goedecke, Kim A. Nguyen, Clement Kufe, Maphoko Masemola, Tinashe Chikowore, Amy E. Mendham, Shane A. Norris, Nigel J. Crowther, Fredrik Karpe, Tommy Olsson, Andre Pascal Kengne, Lisa K. Micklesfield

**Affiliations:** ^1^ Non‐Communicable Diseases Research Unit South African Medical Research Council Cape Town South Africa; ^2^ South African Medical Research Council/WITS Developmental Pathways for Health Research Unit (DPHRU), Department of Paediatrics Faculty of Health Sciences, University of the Witwatersrand Johannesburg South Africa; ^3^ Health through Physical Activity, Lifestyle and Sport Research Centre (HPALS), FIMS International Collaborating Centre of Sports Medicine, Division of Physiological Sciences, Department of Human Biology Faculty of Health Sciences, University of Cape Town Cape Town South Africa; ^4^ Department of Chemical Pathology National Health Laboratory Service and School of Pathology, Faculty of Health Sciences, University of the Witwatersrand Johannesburg South Africa; ^5^ Oxford Centre for Diabetes, Endocrinology and Metabolism (OCDEM), NIHR Oxford Biomedical Research Centre, OUH Foundation Trust University of Oxford Oxford UK; ^6^ Department of Public Health and Clinical Medicine Medicine, Umeå University Umeå Sweden

**Keywords:** metabolic syndrome, obesity, risk stratification, sub‐Saharan African cohort

## Abstract

**Aims:**

To determine the waist circumference (WC) thresholds for the prediction of incident dysglycaemia and type 2 diabetes (T2D) in Black South African (SA) men and women and to compare these to the advocated International Diabetes Federation (IDF) Europid thresholds.

**Materials and Methods:**

In this prospective study, Black SA men (n = 502) and women (n = 527) from the Middle‐aged Sowetan Cohort study who had normal or impaired fasting glucose at baseline (2011‐2015) were followed up until 2017 to 2018. Baseline measurements included anthropometry, blood pressure and fasting glucose, HDL cholesterol and triglyceride concentrations. At follow‐up, glucose tolerance was assessed using an oral glucose tolerance test. The Youden index was used to determine the optimal threshold of WC to predict incident dysglycaemia and T2D.

**Results:**

In men, the optimal WC threshold was 96.8 cm for both dysglycaemia and T2D (sensitivity: 56% and 70%; specificity: 74% and 70%, respectively), and had higher specificity (*P* < 0.001) than the IDF threshold of 94 cm. In women, the optimal WC threshold for incident dysglycaemia was 91.8 cm (sensitivity 86%, specificity 37%) and for T2D it was 95.8 cm (sensitivity 85%, specificity 45%), which had lower sensitivity, but higher specificity to predict incident dysglycaemia and T2D than the IDF threshold of 80 cm (sensitivity: 97% and 100%; specificity: 12% and 11%, respectively)).

**Conclusions:**

We show for the first time using prospective cohort data from Africa that the IDF Europid WC thresholds are not appropriate for an African population, and show that African‐specific WC thresholds perform better than the IDF Europid thresholds to predict incident dysglycaemia and T2D.

## 1 INTRODUCTION

The global prevalence of type 2 diabetes (T2D) is increasing, with sub‐Saharan Africa (SSA) having the highest projected relative rates of increase.^1^ The burden of T2D in SSA is reflected by the high estimated T2D‐associated deaths (~312 000 deaths in 2017), with 73% of these being in people under the age of 60 years, a higher proportion than any other region in the world.^1^ Within SSA, South Africa (SA) has the highest number of people with T2D,^1^ and T2D was the second leading cause of death in SA in 2016 (5.5% of deaths), and the highest amongst women (7.2% of deaths).^2^ Notably, SSA has the highest proportion (59.7%) of people with undiagnosed T2D.^1^ Accordingly, risk stratification that is accessible and cost‐effective is essential for the early detection of T2D to prevent or delay the progression of the disease.

Obesity, in particular central obesity, is an important risk factor for T2D.^3^, ^4^ Although imaging techniques are more accurate measures of total and central adiposity, they are not practical or affordable for routine practice and population‐based risk stratification. Accordingly, anthropometric measures are used as surrogate markers for risk stratification for T2D. Body mass index (BMI) is the most commonly used proxy of total adiposity, while waist circumference (WC) is most often used as a proxy for central adiposity.^4^, ^5^, ^6^

Waist circumference represents the sum of abdominal visceral (VAT) and subcutaneous adipose tissue (SAT), with VAT being the most significant determinant of T2D.^7^, ^8^ However, we and others have shown that for the same level of WC, Black Africans have less VAT than their white European counterparts.^9^, ^10^, ^11^ Accordingly, the WC threshold used for defining risk for T2D may differ in Black Africans. Indeed, both the World Health Organization (WHO) and the International Diabetes Federation (IDF) acknowledge that optimal thresholds for abdominal obesity vary across different ethnicities and population groups.^6^, ^12^ Although several studies in SSA have been undertaken to identify WC thresholds for risk, these have all been cross‐sectional and relied on metabolic syndrome (MetS; excluding WC) as the outcome.^13^, ^14^, ^15^, ^16^ As there is no consensus on an appropriate WC threshold for Black Africans, the IDF has recommended the use of Europid thresholds (≥80 cm in women and ≥94 cm in men) for SSA.^6^ Prospective studies are therefore required to identify the optimal WC thresholds that identify incident T2D in Black African men and women.

While WC is regarded as a useful primary screening tool for T2D, it is also a key feature of MetS, which is also typically used in risk prediction for T2D and cardiovascular diseases.^6^ MetS represents a cluster of risk factors that occur together more often than by chance alone, and in addition to WC, include elevated blood pressure, fasting glucose and triglycerides, and low fasting HDL cholesterol concentrations.^6^ However, it is not clear whether including these additional MetS risk factors improves the discriminatory ability to predict T2D in African men and women when compared to WC alone.

The aim of this study, therefore, was to determine the WC thresholds for the prediction of incident dysglycaemia (prediabetes and T2D) and T2D in Black SA men and women, and to compare these to the advocated Europid thresholds, as defined by the IDF. A secondary aim was to determine if the derived WC thresholds for the prediction of incident dysglycaemia and T2D performed similarly to the presence of MetS in Black SA men and women.

## 2 MATERIALS AND METHODS

### 2.1 Design, study population and setting

**FIGURE 1:**
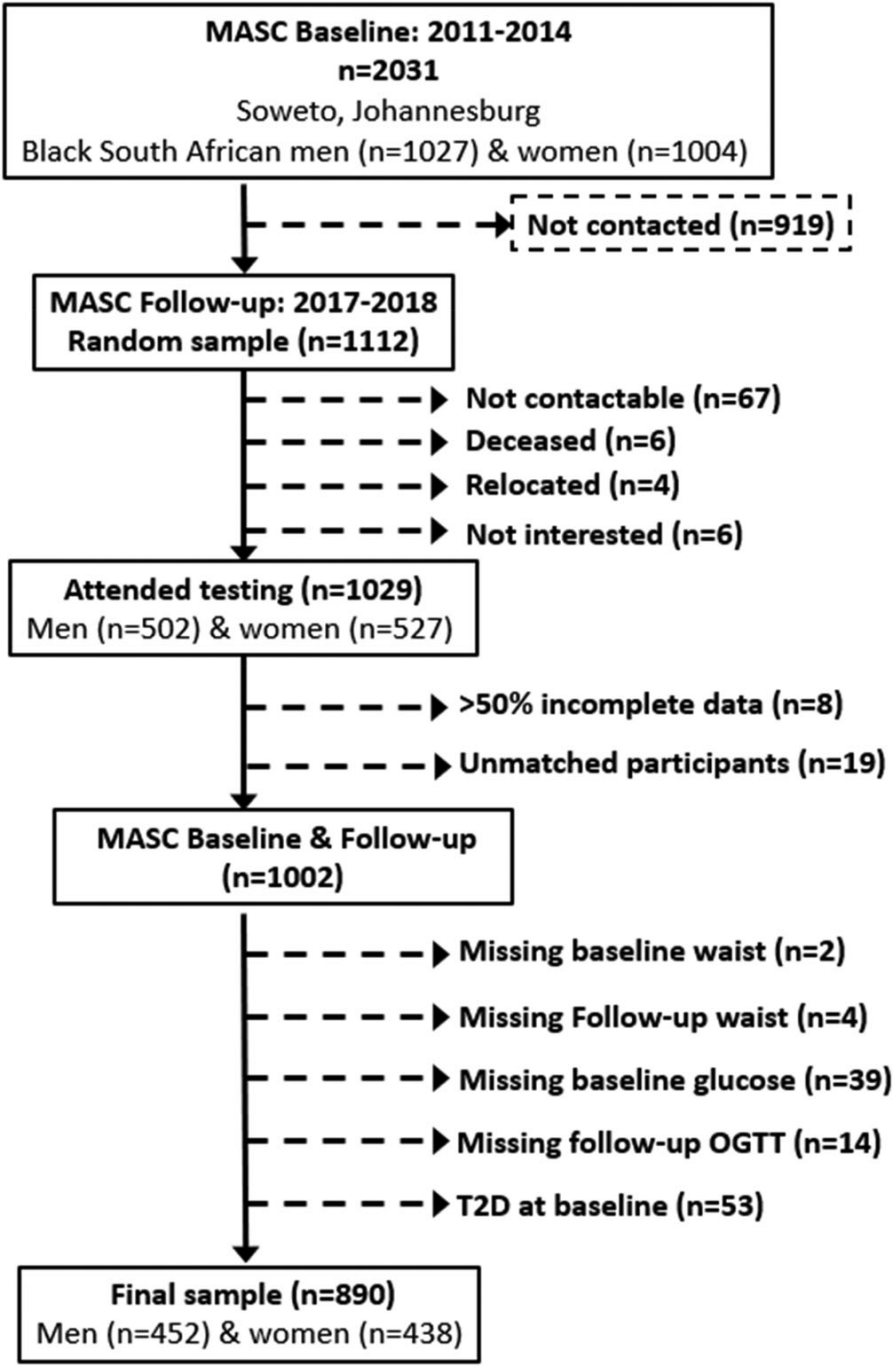
Consort diagram for the Middle‐aged Sowetan Cohort (MASC) waist circumference study

Baseline data collection was part of the African WITS‐INDEPTH Partnerships for Genomic Research (AWI‐Gen) Collaborative Centre, which is a Human Heredity and Health in Africa (H3A) Consortium study,^17^, ^18^ and included 1027 men and 1004 women aged 40 to 60 years living in Soweto, from which the participants in the Middle‐aged Soweto Cohort (MASC) study were randomly selected (n = 1112). The MASC study is a longitudinal study of Black SA men and women residing in Soweto, South Africa, on whom baseline data were collected between 2011 and 2015, and again between January 2017 and August 2018 (Figure 1). Data in this study were collected from a sample of 1029 participants (502 men and 527 women) who were representative of the AWI‐Gen sample and did not differ in terms of age, sex, sociodemographic or lifestyle factors from the main cohort (Supplementary Table S1). Only participants with normal fasting glucose (NFG) or impaired fasting glucose (IFG), and anthropometric measures at baseline, as well as measures of glycaemia from an oral glucose tolerance test at follow‐up, were included in this analysis (Figure 1). Complete data were available for 890 participants (452 men and 438 women).

The study was conducted in accordance with the Declaration of Helsinki and was approved by the Human Research Ethics Committee (Medical) of the University of the Witwatersrand (clearance certificate no. M160604 and M160975). Prior to inclusion in the study, all the procedures and possible risks were explained to the participants who then provided signed consent.

### 2.2 Sociodemographic and health questionnaires

The same interviewer‐administered questionnaire was completed at both time points and included age, current employment (employed/not employed), and highest educational level attained (no formal schooling/elementary school, secondary school, tertiary education). In addition, participants were asked to bring all other current medications, including diabetes medications, to the testing facility for recording and verification. Participants were classified as current smokers/non‐smokers and current alcohol consumers/non‐consumers.

### 2.3 Anthropometry and blood pressure

Weight was measured to the nearest 0.1 kg using a digital scale (model: TBF‐410; Tanita Corporation, Arlington Heights, Illinois). Height was measured to the nearest 0.1 cm using a wall‐mounted stadiometer (Holtain, Crymych, UK). WC and hip circumference were measured to the nearest 0.1 cm with a nonstretchable tape. WC was measured in the mid‐axillary line at the midpoint between the lower margin of the last palpable rib and the top of the iliac crest at the end of normal expiration, and hip circumference was measured at the greatest protrusion of the buttocks.^12^

Systolic and diastolic blood pressure were measured on the left arm using a digital blood pressure monitor (Omron M6, Kyoto, Japan) and appropriate cuffs. After the participant had been seated for at least 5 minutes, three blood pressure readings were taken at 2‐minute intervals. For each participant, the average of the second and third readings was used in the analyses.

### 2.4 Blood sampling and biochemistry

At both baseline and follow‐up, blood samples were drawn after an overnight fast (10‐12 hours) for the measurement of plasma glucose and serum lipid (total cholesterol, HDL cholesterol, LDL cholesterol and triglyceride) concentrations. In the follow‐up sample only, participants then completed a standard 2‐hour oral glucose tolerance test (OGTT). After ingestion of 75 g anhydrous glucose in 250 mL water within 5 minutes, blood samples were drawn at 30‐minute intervals for 2 hours for the subsequent determination of plasma glucose concentrations. Participants with known diabetes and/or those with fasting blood glucose ≥11.1 mmol/L (ACCU‐CHEK®; MedNet GmbH, Munster, Germany) did not complete the OGTT.

Serum lipid concentrations at baseline and plasma glucose concentrations at baseline and follow‐up were measured on the Randox RX Daytona Chemistry Analyser using enzymatic methods (Randox Laboratories Ltd, Crumlin, UK). LDL cholesterol concentrations were calculated using the Friedewald equation.^19^

### 2.5 Glucose tolerance and MetS classification

Glucose tolerance was defined based on the WHO criteria.^20^ At baseline, only fasting glucose samples were available, hence the participants were classified as having NFG if fasting glucose was <6.1 mmol/L, IFG if fasting glucose was 6.1 to 6.9 mmol/L, or T2D if fasting glucose was ≥7 mmol/L and/or if taking T2D medications. Only those with NFG or IFG at baseline were included in this study. At follow‐up, glucose tolerance was defined based on both fasting and 2‐hour OGTT results as follows: normal glucose tolerance if fasting glucose was <6.1 mmol/L and 2‐hour post glucose load was <7.8 mmol/L; IFG (as defined above); impaired glucose tolerance (IGT) if 2‐hour post glucose load was 7.8 to 11.0 mmol/L; and T2D if fasting glucose was >7.0 mmol/L and/or 2‐hour post glucose load ≥11.1 mmol/L. Participants who were taking diabetes medications were classified with T2D. At follow‐up, dysglycaemia, which encompasses both prediabetes and diabetes, was defined as IFG and/or IGT and/or T2D.

Presence of MetS was based on the 2009 harmonized criteria.^6^ Participants with three or more of the following components were classified as having MetS: (i) elevated WC (≥94 cm in men or ≥ 80 cm in women); (ii) elevated fasting triglycerides (≥1.7 mmol/L); (iii) reduced fasting HDL cholesterol (<1.0 mmol/L in men or < 1.3 mmol/L in women); (iv) elevated blood pressure (≥130 mmHg for systolic and/or ≥ 85 mmHg for diastolic blood pressure and/or using blood pressure medication); (v) elevated fasting glucose (≥5.6 mmol/L and/or using diabetes medication).

### 2.6 Statistics

Data analysis was conducted in STATA SE Version 15 (StataCorp, College Station, Texas). Normality of the data was assessed using Shapiro‐Wilks test. As all the descriptive variables were skewed, continuous variables are presented as median (25‐75th percentiles) and categorical variables are presented as frequencies and percentages. Wilcoxon‐Mann‐Whitney tests and chi‐squared tests were used to compare continuous and categorical variables between men and women, respectively. Receiver‐operating characteristic (ROC) area under the curves (AUCs) were used to assess and compare the ability of baseline WC to predict incident dysglycaemia (prediabetes or T2D) and T2D at follow‐up. The standard AUC analysis was used as 46% of the participants were diagnosed with T2D at the follow‐up visit, which precluded time‐to‐event analysis. For the prediction of incident dysglycaemia, only those with NFG at baseline were included in the analysis, whereas for the prediction of incident T2D, those with NFG and IFG at baseline were included in the analysis. Optimal WC thresholds to predict incident dysglycaemia and T2D were determined using Youden's index in men and women separately.^21^ The prognostic performance of the WC thresholds derived in this longitudinal study were assessed alongside the IDF‐defined threshold, as well as WC thresholds defined in other SA and African cross‐sectional studies that have been used to predict the presence of at least two components of MetS, excluding WC.^22^ These studies were used as comparators as, to our knowledge, there are no other studies that have previously defined WC thresholds for predicting incident T2D in Africa. McNemar's test was used to compare the sensitivities and specificities of the derived threshold compared to the recommended IDF thresholds.^23^ Finally, we determined whether including additional MetS risk factors together with the derived WC threshold improved the prediction of incident dysglycaemia and T2D compared to the derived WC thresholds alone.

## 3 RESULTS

### 3.1 Participant characteristics

At baseline, the sample were middle‐aged (~50 years), with men being slightly older than women (Table 1). Women had significantly higher BMIs and a greater proportion of women compared to men were classified with overweight or obesity (88.6% vs. 47.4%; *P* < 0.001 [Table 1]). Accordingly, WC was higher, but waist‐to‐hip ratio was lower in women compared to men. Fasting glucose concentrations were higher in men compared to women, but women had higher triglyceride, total cholesterol and LDL cholesterol concentrations compared to men, while HDL cholesterol concentrations and systolic and diastolic blood pressure did not differ. Although the prevalence of MetS did not differ significantly between men and women, a greater proportion of women had elevated WC, while a greater proportion of men had elevated fasting glucose and reduced HDL cholesterol concentrations.

**TABLE 1:** Characteristics of men and women at baseline

	Men (n = 452)	Women (n = 438)	*P* value
Age, years	50 (45‐55)	49 (45‐54)	0.017
Anthropometry			
BMI, kg/m^2^	24.5 (20.8‐29.1)	32.8 (28.5‐37.3)	<0.001
Waist circumference, cm	88.1 (78.0‐100.0)	98.1 (89.5‐107.0)	<0.001
Hip circumference, cm	99.4 (91.2‐106.1)	117.0 (108.5‐126.0)	<0.001
Waist‐to‐hip ratio	0.90 (0.85‐0.95)	0.84 (0.78‐0.89)	<0.001
BMI categories, % (n)			
Underweight	10.0 (46)	0.5 (2)	<0.001
Normal weight	42.6 (196)	11.0 (48)	
Overweight	26.5 (122)	21.9 (96)	
Obese	20.9 (96)	66.7 (292)	
Biochemistry			
Fasting glucose, mmol/L	5.1 (4.7‐5.4)	4.8 (4.5‐5.2)	<0.001
Triglycerides, mmol/L	0.9 (0.6‐1.3)	1.0 (0.7‐1.4)	<0.001
Total cholesterol, mmol/L	4.1 (3.4‐4.8)	4.5 (3.8‐5.2)	<0.001
HDL cholesterol, mmol/L	1.2 (0.9‐1.5)	1.2 (1.0‐1.5)	0.063
LDL cholesterol, mmol/L	2.5 (1.8‐3.1)	2.7 (2.2‐3.3)	<0.001
Blood pressure			
Systolic, mmHg	129.0 (117.0‐144.0)	128.5 (117.0‐141.5)	0.518
Diastolic, mmHg	88.8 (79.5‐96.5)	87.5 (79.0‐96.0)	0.357
Metabolic syndrome (JIS)			
Elevated WC, % (n)	37.6 (170)	90.4 (396)	<0.001
Elevated fasting glucose, % (n)	15.9 (72)	10.5 (46)	0.017
Elevated triglycerides, % (n)	13.1 (59)	14.4 (63)	0.564
Reduced HDL cholesterol, % (n)	69.3 (313)	46.1 (202)	<0.001
Elevated blood pressure, % (n)	61.3 (279)	59.6 (261)	0.514
MetS, % (n)	30.3 (137)	35.4 (155)	0.107

*Note*: Values are median (25‐75th percentile).

Abbreviations: BMI, body mass index; JIS, Joint Interim Statement; MetS, metabolic syndrome.

*Note*: MetS was determined using the JIS^6^ and defined as meeting any three of the following criteria: elevated waist circumference (≥80 cm in women; ≥94 cm in men); elevated blood glucose (≥5.6 mmol/L and/or using diabetes medication); elevated fasting triglycerides (≥1.7 mmol/L); reduced fasting HDL cholesterol (<1.0 mmol/L in men, <1.3 mmol/L in women); elevated blood pressure (≥135/85 mmHg and/or using antihypertension medication).

The median (25‐75th percentile) follow‐up times were 3.1 (2.9‐3.5) years in men and 4.8 (4.0‐5.5) years in women. Of the 430 men and 421 women with NFG at baseline, 73 men and 101 women developed dysglycaemia at follow‐up, resulting in a cumulative incidence of 17.0% (95% confidence incidence [CI] 13.4‐28.1) in men and 24.0% (95% CI 19.9‐41.1) in women. Of the 452 men and 438 women with NFG or IFG at baseline, 20 men and 47 women developed T2D at follow‐up, resulting in a cumulative incidence of T2D of 4.4% (95% CI 2.5‐5.9) in men and 10.7% (95% CI 7.8‐16.8) in women.

### 3.2 Performance of different WC thresholds to predict incident dysglycaemia and T2D

**FIGURE 2:**
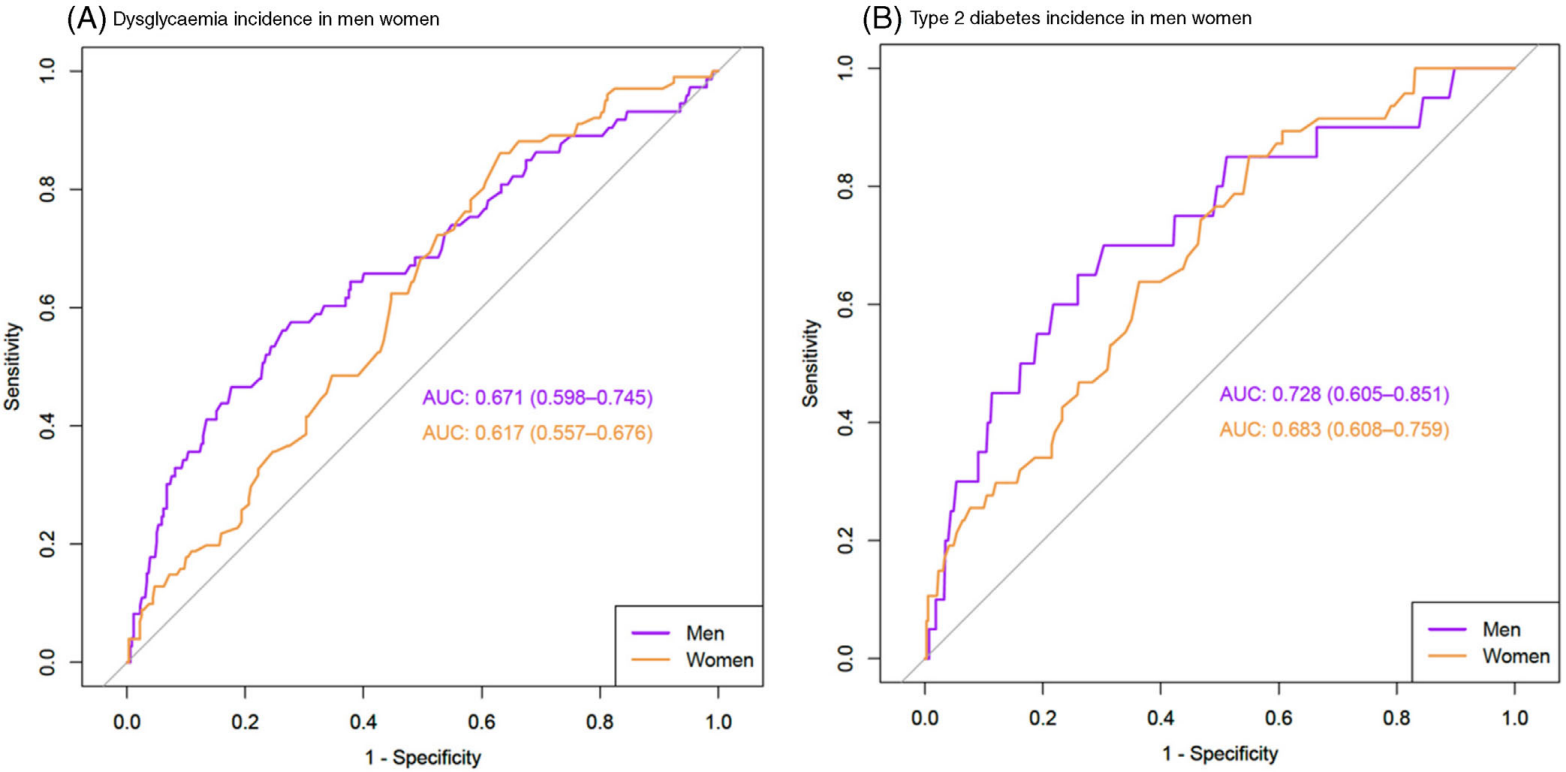
Receiver‐operating characteristic area under the curve (AUC) of waist circumference to predict A, incident dysglycaemia and B, type 2 diabetes in Black African men and women

The ROC analyses for WC to predict incident dysglycaemia and T2D in men and women are presented in Figure 2. WC showed acceptable discrimination to predict dysglycaemia and T2D in men and women, with the AUCs being higher in men than women (Figure 2). Based on the Youden's index, the optimal WC thresholds to predict incident dysglycaemia in men and women were 96.8 cm and 91.8 cm, respectively (Table 2). In men, this threshold was similar to the IDF threshold of 94 cm, and accordingly had similar sensitivity (*P* = 0.250), but significantly higher specificity (*P* < 0.001). However, the threshold of 96.8 cm was higher than those derived from cross‐sectional studies of other African populations to detect MetS (84‐90 cm), with a resultant lower sensitivity, but higher specificity.

**TABLE 2:** Performance measures of different waist circumference thresholds to predict incident dysglycaemia in Black African men and women

Reference	Cut‐off, cm	Sensitivity (95%CI)	Specificity (95%CI)	PPV (95%CI)	NPV (95%CI)	Youden index (95% CI)
Men						
Current study	96.8	0.56 (0.44‐0.68)	0.74^#^ (0.69‐0.78)	0.30 (0.23‐0.39)	0.89 (0.85‐0.92)	0.30 (0.13‐0.46)
IDF ^6^	94	0.59 (0.47‐0.70)	0.68^#^ (0.63‐0.73)	0.27 (0.21‐0.35)	0.89 (0.85‐0.92)	0.27 (0.10‐0.43)
Matsha et al.^13^	90	0.66 (0.54‐0.76)	0.59 (0.54‐0.64)	0.25 (0.19‐0.31)	0.89 (0.85‐0.93)	0.25 (0.07‐0.40)
Ekoru et al.^15^	81	0.82 (0.72‐0.90)	0.35 (0.30‐0.40)	0.20 (0.16‐0.26)	0.91 (0.84‐0.95)	0.17 (0.01‐0.30)
Motala et al.^16^	86	0.68 (0.57‐0.79)	0.48 (0.43‐0.53)	0.21 (0.16‐0.27)	0.88 (0.83‐0.92)	0.17 (0.01‐0.32)
Peer et al.^24^	83.9	0.74 (0.62‐0.84)	0.45 (0.40‐ 0.50)	0.22 (0.17‐0.27)	0.89 (0.84‐0.94)	0.19 (0.02‐0.34)
MetS	96	0.44 (0.32‐0.56)	0.77 (0.72‐0.81)	0.28 (0.20‐0.37)	0.87 (0.83‐0.90)	0.21 (0.04‐0.37)
Women						
Current study	91.8	0.86* (0.78‐0.92)	0.37^#^ (0.32‐0.42)	0.30 (0.25‐0.36)	0.89 (0.83‐0.94)	0.23 (0.09‐0.35)
IDF ^6^	80	0.97* (0.92‐0.99)	0.12^#^ (0.09‐0.16)	0.26 (0.22‐0.31)	0.93 (0.81‐0.99)	0.09 (0.01‐0.16)
Matsha et al.^13^	90	0.88 (0.80‐0.94)	0.31 (0.26‐0.36)	0.29 (0.24‐0.34)	0.89 (0.82‐0.94)	0.19 (0.06‐0.30)
Ekoru et al.^15^	81	0.97 (0.92‐0.99)	0.14 (0.10‐ 0.18)	0.26 (0.22‐0.31)	0.94 (0.83‐0.99)	0.11 (0.02‐0.18)
Motala et al.^16^	92	0.86 (0.78‐0.92)	0.37 (0.32‐ 0.42)	0.30 (0.25‐0.36)	0.89 (0.83‐0.94)	0.23 (0.09‐0.35)
Crowther et al.^14^	91.5	0.86 (0.78‐0.92)	0.36 (0.30‐ 0.41)	0.30 (0.25‐0.35)	0.89 (0.82‐0.94)	0.22 (0.08‐0.33)
Peer et al.^24^	94	0.76 (0.67‐0.84)	0.43 (0.37‐0.48)	0.30 (0.24‐0.36)	0.85 (0.79‐0.90)	0.19 (0.04‐0.33)
MetS	96	0.38 (0.28‐0.48)	0.80 (0.75‐0.84)	0.37 (0.28‐0.47)	0.80 (0.75‐0.84)	0.17 (0.03‐0.32)

Abbreviations: FN, false‐negative; FP, false‐positive; IDF, International Diabetes Federation; MetS, metabolic syndrome; NPV, negative predictive value; PPV, positive predictive value; TN, true‐negative; TP, true‐positive.

*Note*: Dysglycaemia was defined as impaired fasting glucose and/or impaired glucose tolerance or type 2 diabetes. Current study thresholds were based on the Youden index. Sensitivity = TP/(TP + FN). Specificity = TN/(TN + FP). PPV = TP/(TP + FP). NPV = sensitivity/1‐specificity. Youden's index = (sensitivity + specificity)– 1. MetS was defined using a waist circumference threshold of 96 cm for men and women derived from this study. *P = 0.001 for the difference in the sensitivity between the derived and IDF thresholds; ^#^ *P* < 0.001 for the difference between in the specificity between the derived and IDF thresholds.

In women, the threshold of 91.8 cm to predict incident dysglycaemia was higher than the IDF‐recommended threshold of 80 cm but was similar to most thresholds from other African studies to detect MetS (Table 2). Although the sensitivity was lower than the IDF threshold (0.86 vs. 0.97; *P* = 0.001), the specificity (0.37 vs. 0.12; *P* < 0.001) was higher using the derived threshold of 91.8 cm.

The optimal WC threshold to predict incident T2D in men (Table 3) was the same as that for dysglycaemia (96.8 cm) and consequently all the performance variables are the same as those reported for dysglycaemia in Table 2. In contrast, the optimal WC threshold to predict incident T2D in women was 95.8 cm (Table 3), which is similar to the threshold for dysglycaemia and T2D in men, but higher than the threshold for dysglycaemia (91.8 cm), and the IDF cut‐off point (80 cm) and most other thresholds derived to detect MetS in African women (81‐94 cm). Although the sensitivity of the optimal threshold of 95.8 cm was lower than for the IDF threshold (0.85 vs. 1.00; *P* = 0.016) and other African studies (0.87‐1.00), the specificity was significantly higher (0.45 vs. 0.11 and 0.12‐0.40; *P* < 0.001).

**TABLE 3:** Performance measures of different waist circumference thresholds to predict incident type 2 diabetes in Black African men and women

Reference	Cut‐off, cm	Sensitivity (95% CI)	Specificity (95% CI)	PPV (95% CI)	NPV (95% CI)	Youden index (95% CI)
Men						
Current study	96.8	0.70 (0.46‐0.88)	0.70^#^ (0.65‐0.74)	0.10 (0.05‐0.16)	0.98 (0.96‐ 0.99)	0.40 (0.11‐ 0.62)
IDF ^6^	94	0.70 (0.46‐0.88)	0.64^#^ (0.59‐0.68)	0.08 (0.05‐0.13)	0.98 (0.95‐ 0.99)	0.34 (0.05‐0.57)
Matsha et al.^13^	90	0.75 (0.51‐0.91)	0.55 (0.50‐0.59)	0.07 (0.04‐0.11)	0.98 (0.95‐ 0.99)	0.30 (0.01‐0.51)
Ekoru et al.^15^	81	0.90 (0.68‐0.99)	0.32 (0.27‐0.36)	0.06 (0.03‐0.09)	0.99 (0.95‐ 1.00)	0.22 (‐0.04‐0.35)
Motala et al.^16^	86	0.85 (0.62‐0.97)	0.45 (0.40‐0.50)	0.07 (0.04‐0.10)	0.98 (0.96‐ 1.00)	0.30 (0.02‐0.46)
Peer et al.^24^	83.9	0.85 (0.62‐0.87)	0.41 (0.37‐0.46)	0.06 (0.04‐0.10)	0.98 (0.95‐ 1.00)	0.26 (‐0.01‐0.43)
MetS	96	0.45 (0.23‐0.68)	0.72 (0.68‐0.77)	0.07 (0.03‐0.13)	0.97 (0.94‐0.98)	0.17 (‐0.09‐0.45)
Women						
Current study	95.8	0.85* (0.72‐0.94)	0.45^#^ (0.40‐0.50)	0.16 (0.11‐0.21)	0.96 (0.92 0.98)	0.30 (0.12‐ 0.44)
IDF ^6^	80	1.00* (0.92‐1.00)	0.11^#^ (0.08‐0.14)	0.12 (0.09‐0.15)	1.00 (0.92‐ 1.00)	0.11 (0.01‐0.14)
Matsha et al.^13^	90	0.91 (0.80‐0.98)	0.28 (0.23‐0.32)	0.13 (0.10‐0.17)	0.96 (0.91‐ 0.99)	0.19 (0.03‐0.30)
Ekoru et al.^15^	81	1.00 (0.92‐1.00)	0.12 (0.09‐0.16)	0.12 (0.09‐0.16)	1.00 (0.93‐ 1.00)	0.12 (0.02‐0.16)
Motala et al.^16^	92	0.91 (0.80‐0.98)	0.33 (0.29‐0.38)	0.14 (0.10‐0.19)	0.97 (0.93‐ 0.99)	0.24 (0.08‐0.36)
Crowther et al.^14^	91.5	0.91 (0.80‐0.98)	0.32 (0.28‐0.37)	0.14 (0.10‐0.18)	0.97 (0.92‐ 0.99)	0.24 (0.07‐0.35)
Peer et al.^24^	94	0.87 (0.74‐0.95)	0.40 (0.36‐0.45)	0.15 (0.11‐0.20)	0.96 (0.92‐0.99)	0.28 (0.10‐0.41)
MetS	96	0.49 (0.34‐0.64)	0.76 (0.72‐0.80)	0.20 (0.13‐0.28)	0.93 (0.89‐0.95)	0.25 (0.06‐0.44)

Abbreviations: FN, false negative; FP, false‐positive; IDF, International Diabetes Federation; MetS, metabolic syndrome; NPV, negative predictive value; PPV, positive predictive value; TN, true‐negative; TP, true‐positive.

*Note*: Current study thresholds were based on the Youden index. Sensitivity = TP/(TP + FN). Specificity = TN/(TN + FP). PPV = TP/(TP + FP). NPV = sensitivity/1‐specificity. Youden's index = (sensitivity + specificity)– 1. MetS was defined using a waist circumference threshold of 96 cm for men and women derived from this study. *P = 0.016 for the difference in the sensitivity between the derived and IDF thresholds. ^#^ *P* < 0.001 for the difference in the specificity between the derived and IDF thresholds.

### 3.3 Comparative ability of derived WC thresholds versus MetS to predict incident dysglycaemia and T2D

We then determined whether including additional MetS risk factors together with the derived WC thresholds improved the prediction of incident dysglycaemia and T2D compared to the derived WC thresholds alone. In Tables 2 and 3, we showed that the threshold to predict incident dysglycaemia and T2D in men, and T2D in women were similar (~96 cm). Other African studies that have examined thresholds to detect MetS have also suggested similar thresholds for men and women.^13^, ^15^, ^16^ Thus, we used the WC threshold of 96 cm in both men and women as the WC component of MetS and compared this to the WC threshold alone, to predict incident dysglycaemia and T2D in men and women (Tables 2 and 3). Despite including additional risk factors, MetS had lower sensitivity, but similar specificity compared to the optimal WC threshold of 96.8 cm to predict incident dysglycaemia and T2D in men (Tables 2 and 3). In contrast, the sensitivity of MetS using a WC threshold of 96 cm to predict both incident dysglycaemia and T2D in women was lower than for the derived thresholds alone, while the specificity was higher (Tables 2 and 3).

## 4 DISCUSSION

This is the first prospective study to examine WC thresholds to predict incident dysglycaemia and T2D in an African population and showed that the optimal thresholds differed from those in European populations. The optimal thresholds to predict incident dysglycaemia and T2D in Black SA men were 96.8 cm for both outcomes and in women they were 91.8 and 95.8 cm, respectively. Importantly, these African‐specific WC thresholds had significantly higher specificity than the IDF Europid thresholds.

The WC thresholds to predict incident dysglycaemia and T2D in women were higher than the IDF threshold of 80 cm. This is consistent with the findings of several cross‐sectional studies in SA that detected MetS, defined as at least two components of MetS excluding WC, and reported optimal thresholds of 90 to 94 cm.^13^, ^14^, ^16^, ^24^ We showed that in women the thresholds of 91.8 and 95.8 cm had lower sensitivity and higher specificity than the IDF threshold to predict incident dysglycaemia and T2D, respectively. The specificity of the IDF threshold was as low as 0.11, suggesting 89% of Black SA women who will remain free of dysglycaemia or T2D over time may be incorrectly classified among those who will go on to develop the conditions if this threshold was used alone as a risk screening tool.

Nonetheless, the devised African‐specific threshold still had low specificity for incident dysglycaemia and T2D (0.37 and 0.45, respectively) in Black SA women. This was also lower than that reported in Black SA men using the derived threshold of 96.8 cm (specificity ~0.70). The poor discriminatory ability of the WC thresholds to predict incident dysglycaemia and T2D in women may be due to the women's high levels of obesity (66.7% vs. 20.9%) and central obesity (90% WC >80 cm vs. 37.6% WC >94 cm) compared to the men in this sample. However, this is representative of men and women of this age group in South Africa.^25^ Further, these discrepant findings may be explained by the stronger association between total and central adiposity and T2D risk in Black SA men compared to Black SA women.^26^ WC incorporates both VAT and SAT, and it has been shown in Black SA people and African‐born Black people living in the United States that for the same WC, women have less VAT and more abdominal SAT than men.^26^, ^27^ Indeed, in the SA cohort, the VAT: SAT ratio was twofold higher in men versus women (0.50 vs. 0.24),^25^ whereas in the study in African‐born Black people living in the United States, the VAT: SAT ratio was threefold higher in men than women (~0.91 vs. 0.31).^27^ Higher abdominal SAT for every level of VAT explained the higher WC cut‐off point required for predicting insulin resistance in African‐born Black women living in the United States compared to their male counterparts (96 cm vs. 91 cm).^27^ In addition, Black African women have greater gluteofemoral fat than their male counterparts, which is associated with reduced cardiometabolic risk, independent of VAT.^28^ Indeed, a prospective study in Black SA women showed that VAT and leg fat mass, but not abdominal SAT, predicted the development of T2D.^7^ Accordingly, high levels of abdominal and peripheral SAT in Black SA women may mask the association with VAT and lead to poor discriminatory power of WC to predict incident T2D.

Previous studies from SSA have suggested that the WC threshold of cardiometabolic risk should be similar for African men and women.^13^, ^15^, ^16^ The findings from this study support this recommendation. Indeed, a WC of 96.8 cm predicted both incident dysglycaemia and T2D in men, whilst a very similar WC of 95.8 cm predicted incident T2D in women. However, larger independent longitudinal studies are required to confirm our findings for the SA population. We do agree that WC thresholds are dependent on the underlying obesity prevalence and should be region‐specific.^15^

Another important finding of this study was that when including additional MetS risk factors together with the derived WC threshold to predict incident dysglycaemia and T2D in men, the predictive accuracy did not change. In contrast, in women, the inclusion of the additional MetS risk factors resulted in a decrease in sensitivity (0.85‐0.86 to 0.38‐0.49), but an increase in specificity (0.37‐0.45 to 0.80‐0.76). In both men and women, the most common features of MetS were reduced HDL cholesterol levels and elevated blood pressure. In African populations, reduced HDL cholesterol levels are not necessarily a marker of cardiometabolic risk, and the WHO sex‐based cut‐offs are inappropriate^29^, ^30^, ^31^ while blood pressure is a risk factor for cardiovascular disease rather than T2D. Further, it has been previously shown that MetS and its components, in particular triglyceride and HDL cholesterol levels, are not associated with insulin resistance in Black African women^32^, ^33^, ^34^ and that MetS may not be a good indicator of cardiometabolic risk in Black African populations.^35^, ^36^, ^37^ The time and cost associated with these additional measures is unlikely to offset the reduction in false‐positives associated with WC measures alone in Black SA women. Concomitantly, this highlights the need for future studies to establish accessible and cost‐effective risk biomarkers that have high sensitivity and specificity for the early detection of T2D in SSA.

The major strengths of this study are the prospective design and the diagnosis of incident dysglycaemia and T2D using an OGTT. To date, all studies that have explored thresholds for WC have been cross‐sectional and were designed for identifying the optimal WC cut‐off point for detecting MetS.^13^, ^14^, ^16^, ^24^ We used an OGTT to diagnose T2D at follow‐up, which is considered the “gold standard”, particularly in African populations where fasting glucose and glycated haemoglobin (HbA1c) may perform sub‐optimally.^38^, ^39^, ^40^, ^41^ Another strength of this study is the inclusion of equal numbers of men and women. Most studies in SA have either focused on women only or included small samples of men.^13^, ^14^, ^15^, ^16^, ^24^ Limitations of the study include the relatively small sample size, which precluded the validation of the threshold in a subsample of the participants, the relatively short and different follow‐up periods for men and women (3.1 and 4.8 years, respectively), with the latter precluding sex comparisons in incidence rates. Another limitation is the use of only fasting glucose at baseline, which may have resulted in the inclusion of participants with baseline IGT or T2D based on 2‐hour OGTT results. Indeed, using the follow‐up data for which we have both fasting and 2‐hour glucose results, we showed that in men and women with NFG, 8.4% (n = 34) and 12.1 (n = 45) had IGT, and 1.2% (n = 5) and 1.4% (n = 5) had T2D based on 2‐hour OGTT results, respectively. Future definitive studies should include the following key design elements: a large representative sample of the population; OGTT measures at both baseline and follow‐up; adequate follow‐up time; similar follow‐up times in men and women; accurate time‐to‐event information; and an independent validation cohort.

In conclusion, we show for the first time using prospective cohort data from South Africa that the IDF Europid WC thresholds are not appropriate for an African population and that African‐specific WC thresholds perform better than the IDF Europid thresholds in predicting incident dysglycaemia and T2D in Black SA men and women. These findings verify existing evidence from cross‐sectional studies showing suboptimal performance of currently recommended WC thresholds in African populations,^13^, ^15^, ^16^ but require validation in an independent longitudinal cohort from Africa.

## CONFLICT OF INTEREST

The authors declare no competing financial interests.

## AUTHOR CONTRIBUTIONS

The authors confirm contribution to the paper as follows: study conception and design: Julia H. Goedecke and Lisa K. Micklesfield; data collection: Clement Kufe, Maphoko Masemola, Tinashe Chikowore; data analysis: Julia H. Goedecke, Kim Nguyen, Andre Pascal Kengne; interpretation of results: Julia H. Goedecke, Kim Nguyen, Andre Pascal Kengne, Amy E. Mendham, Shane A. Norris, Nigel J. Crowther, Fredrik Karpe, Tommy Olsson, Lisa K Micklesfield; draft manuscript preparation: Julia H. Goedecke. All authors critically reviewed the results and approved the final version of the manuscript. Julia H. Goedecke is the guarantor and takes full responsibility for the contents of the manuscript.

### PEER REVIEW

The peer review history for this article is available at https://publons.com/publon/10.1111/dom.14655.

## Supporting information


**Supplementary Table S1.** Baseline comparison of the characteristics of those selected and not selected for follow‐up from the MASC Cohort

## Data Availability

The data that support the findings of this study are available from the corresponding author upon reasonable request.
