# Supplementary material for: Waist circumference thresholds predicting incident dysglycaemia and type 2 diabetes in Black African men and women
Source: Diabetes Obes Metab. 2022 Feb 10;24(5):918–27. doi: 10.1111/dom.14655 (PMC9305761; doi:10.1111/dom.14655)
Supplement: Supplementary file 1 — Supplementary Table S1. Baseline comparison of the characteristics of those selected and not selected for follow‐up from the MASC Cohort [file DOM-24-918-s001.docx]

**Supplementary Table S1.** Baseline comparison of the characteristics of those selected and not selected for follow-up from the MASC Cohort

| **Variable** | **Not selected for follow-up** | **Selected for follow-up** | **P value** |
| --- | --- | --- | --- |
| n | 1018 | 1002 |  |
| Females (n(%)) | 491 (48.3) | 501 (50.0) | 0.439 |
| Age (years) | 49 (44-54) | 49 (44-54) | 0.062 |
| Employed (n(%)) | 623 (61.3) | 592 (59.1) | 0.318 |
| Education (years) | 8 (5-11) | 8 (5-12) | 0.581 |
| Socioeconomic status quintile (n(%)) | 3 (2-4) | 3 (2-5) | 0.089 |
| Tobacco use (n(%)) | 424 (41.7) | 399 (39.8) | 0.392 |
| Alcohol use (n(%)) | 374 (36.4) | 354 (35.4) |  |
| Moderate-to-vigorous physical activity (min/wk) | 390 (110-1440) | 420 (120-1300) | 0.998 |
| Menopausal status |  |  | 0.254 |
| Pre-menopausal (n(%)) | 160 (33.3) | 171 (34.5) |  |
| Peri-menopausal (n(%)) | 81 (16.9) | 65 (13.1) |  |
| Post-menopausal (n(%)) | 239 (49.8) | 260 (52.4) |  |

Values are presented as n(%) or median (25-75^th^ percentile)
